# Fibre Bragg Grating Based Strain Sensors: Review of Technology and Applications

**DOI:** 10.3390/s18093115

**Published:** 2018-09-15

**Authors:** Carlo Edoardo Campanella, Antonello Cuccovillo, Clarissa Campanella, Abdulkadir Yurt, Vittorio M. N. Passaro

**Affiliations:** 1 Photonics Research Group, Dipartimento di Ingegneria Elettrica e dell’Informazione, Politecnico di Bari, via E. Orabona n., 4, 70125 Bari, Italy; 2 QOpSyS SRL, Via Matteotti 23, Gioia del Colle, 70023 Bari, Italy; an.cuccovillo@gmail.com (A.C.); clary.campanella@gmail.com (C.C.); Kdryrt@gmail.com (A.Y.)

**Keywords:** optical devices, fibre Bragg grating, optical sensors

## Abstract

Fibre Bragg grating (FBG) strain sensors are not only a very well-established research field, but they are also acquiring a bigger market share due to their sensitivity and low costs. In this paper we review FBG strain sensors with high focus on the underlying physical principles, the interrogation, and the read-out techniques. Particular emphasis is given to recent advances in highly-performing, single head FBG, a category FBG strain sensors belong to. Different sensing schemes are described, including FBG strain sensors based on mode splitting. Their operation principle and performance are reported and compared with the conventional architectures. In conclusion, some advanced applications and key sectors the global fibre-optic strain sensors market are envisaged, as well as the main market players acting in this field.

## 1. Introduction

**Figure 1:**
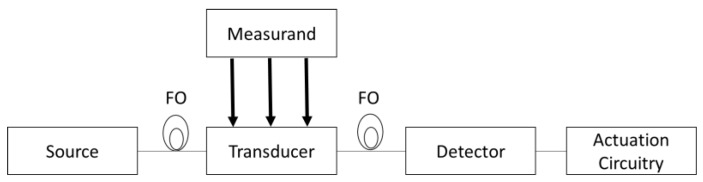
Fiber optic sensor simplified architecture.

The fibre optics communication industry has revolutionized the telecommunication industry through providing more reliable telecommunication links, higher performance, and constantly decreasing bandwidth cost. The technological progress of fibre optic communication has stimulated the development of the fibre optic sensors [1]. Fibre-optic sensors offer advantages over other existing sensing technologies such as increased sensitivity and form factor versatility [2]. The functional peculiarities of fibre optic sensors have been exploited to replace traditional sensors in a wide range of applications including strain, vibration, electric, acoustic and magnetic fields, acceleration, rotation, pressure, temperature, linear and angular position, humidity, viscosity, chemical measurements, and many others. Due to their dielectric property, fibre-optic sensors can be used in harsh environments such as high temperature, high voltage, or in the presence of corrosive materials; in addition, these sensors are compatible with communication systems and are able to perform remote sensing. A simplified architecture of fiber optic sensor is reported in Figure 1. It consists of an optical source that excites the transducer (i.e., the sensitive optical element) through a fiber optic cable (FO). Due to a variation of the measurand, the transducer converts the initial signal of the optical source in another signal having different features. The modificed signal is detected from a detector and, then, processed by the actuation circuity. The actuation circuitry derives the informations about the measurand through a comparison between the initial signal, usually called reference signal, and the signal modified by the transducer. 

The main advantages of fibre-optic sensors include their ability to be lightweight, very small sized, passive, and immune to electromagnetic interference (EMI). Additionally, they require low power and they induce low attenuation as well as they are characterized by high sensitivity, wide bandwidth, and environmental ruggedness. Their main disadvantages are high cost and unfamiliarity to the end user [3,4,5].

Optical sensors based on Fibre Bragg Gratings (FBGs) have acquired a large market share due to a number of advantages: small form factor, lightweight, no need for electrical connections, and the compatibility for non-invasive remote sensing. The FBGs peculiarities of showing high sensitivity, high resolution, and wide dynamic range, as well as their intrinsic immunity to radio frequency interference (RFI) and electromagnetic interference (EMI), and their capability of being interfaced with data communication systems, contributed to their widespread employing in many sensing applications.

Due to FBGs’ high sensitivity to multiple environmental parameters, including physical, chemical, biomedical, and electrical parameters, they are used for structural health monitoring in civil infrastructures, aerospace, energy, and maritime areas, where the information associated to measurands are usually encoded by the Bragg wavelength shift of FBGs.

In this paper, a general review of the FBG strain sensors, interrogation techniques, performance, and their application fields are presented. The investigation begins with the analysis of the measurand (i.e., strain). Strain is a normalized measure of the deformation, which represents the displacement between particles in the matter with respect to a reference length.

The strain is related to the motions of a rigid body, such as translations and rotations, and variation of the shape/size of the matter [6].

From a physical point of view, the strain is described through a tensor quantity, which can be decomposed into a normal and shear component. The normal component takes into account the stretch or compression along the material line elements or fibres, while the shear component is related to the sliding of layers composing the body over each other. If the length of body increases, the normal strain is called tensile strain, if it reduces, the strain is called compressive strain [7].

The engineering strain (or engineering extensional strain or nominal strain) is expressed as the ratio between the total deformation and the initial state. The strain is tensile if the total deformation is positive, while it is compressive if the total deformation is negative. The strain unit is a dimensionless value and it is given by:

(1) $$\varepsilon =\frac{l-L}{L}=\frac{\Delta L}{L}$$

where *ε* is the engineering normal strain, *l* is the final length, and *L* is the initial one. The true shear strain is the change of the angle between two material line elements which are perpendicular into the unperturbed state, while the engineering shear strain is the tangent of that angle.

In the case of fibre-optics strain sensors, the strain applied to the fibre can be seen as the ratio between the total wavelength shift Δλ and the initial wavelength λ. Due to the photo-elastic coefficient that links Δλ/λ with ΔL/L through the relation Δλ/λ = 0.79 ΔL/L [8], for a conventional single mode fibre SMF28, we can write:

(2) $$\frac{\Delta \lambda }{\lambda }=0.79\;\varepsilon$$

It is important to clarify that the cause of the strain is the stress. Differently from the strain, the stress expresses the internal forces that particles exert on each other in a continuous material, while the strain is the consequence of these forces. If the stress exceeds a certain strength limit of the material, it will be permanently deformed or it will change its crystal structure and chemical composition, otherwise the stress is reversible. The stress is defined as the average force per unit area that some particle of the object applies on the adjacent one, across an imaginary surface that separates them. Such as for the strain, the stress can be classified as normal stress whether related to compression and tension, and shear stress whether related to stress parallel to the surface. The stress unit is that of pressure.

After briefly covering the concepts of strain and stress, the main categories of fibre optics strain sensors are presented in the following section, where the FBGs are emphasised for their fundamental role as single-point sensors in which the sensing head is obtained through a periodic modulation of the refractive index.

## 2. Optical Fibre Devices for Strain Sensing

**Figure 2:**
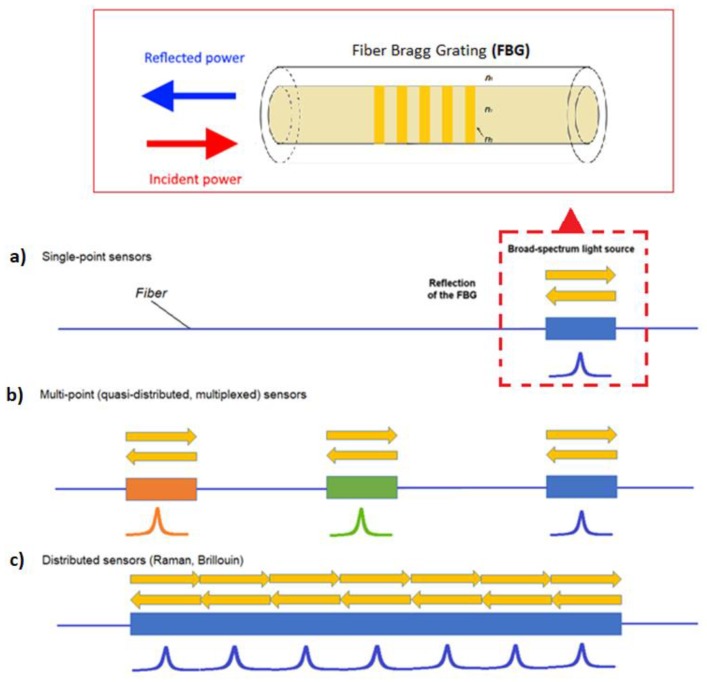
Fibre-optic strain sensing categories: single-point sensors, including (**a**) FBG sensors, (**b**) quasi distribute (multiplexed), and (**c**) distributed sensors.

Fibre optic strain sensors can be classified into three categories: single-point sensors [9], quasi-distributed (multiplexed), and distributed sensors (see Figure 2) [10]. Single-point sensors are small, durable, and highly accurate sensor units, usually attached to a high-bandwidth fibre-optic cable. These single-point sensors can be multiplexed and placed at strategic locations along the fibre to construct a quasi-distributed measurement. Distributed sensing uses the fibre-optical cable to monitor strain across the entire length of the structure under investigation [11,12,13,14,15].

**Figure 3:**
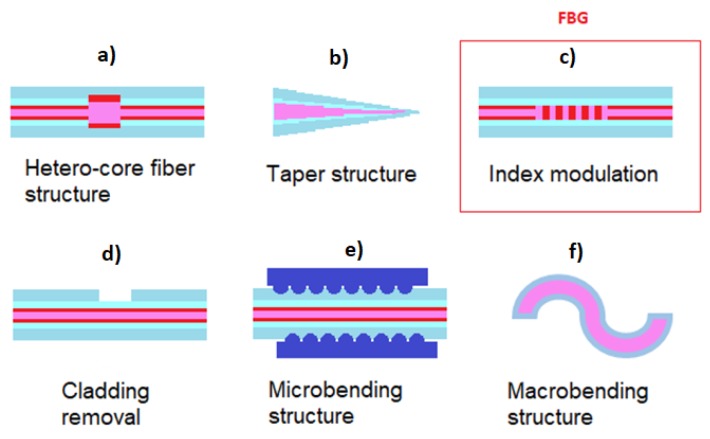
Sensors geometries: (**a**) hetero-core fibre structure, (**b**) taper structure, (**c**) FBG, (**d**) cladding removal, (**e**) micro-bending structure, and (**f**) macro-bending structure.

The FBG strain sensors belong to the category of single-point sensors and preserve the features of small size, durability, and high accuracy. For completeness, other geometries of single-point sensors are reported in Figure 3. The Bragg gratings can be realized using micro-fabrication methods creating refractive index modulation along the beam propagation direction. Besides the FBGs based on refractive index modulation, they can be also realized using hetero-core fibre structures, taper structures, cladding removal, micro-bending structure, and macro-bending structure.

## 3. Key FBG Performance Factors of the Fibre-Optic Sensors

The key performance factors of the FBG strain sensors are generally the same as the fibre-optic sensors. They include the sensitivity, representing the relationship between the variation of the sensor output and the corresponding variation in measurand. In an optoelectronic sensor, the sensitivity is usually defined as the variation of optical power at the receiver, produced by a unit variation of the measured magnitude. A good sensor must have a high sensitivity, i.e., small variations in the magnitude to be measured must correspond to large variations in the output. If the sensor output varies linearly with the magnitude to be measured, then the sensitivity of the sensor can be defined as a “scale factor”. An additional key performance factor is the resolution. It expresses the capability of the sensor to detect small variations in the measurand. It is defined as “the variation in the value of the measurand that causes a variation in the output value equal to the uncertainty of the output itself”. Resolution can be expressed in absolute value, or relative or reduced value. Spatial resolution can be described as the smallest length within which a significant change in the measured magnitude can be detected. Referring to the uncertainty of the output corresponds to considering the slightest variation without any randomness from a given operative point. Often the resolution is indicated with the term “dead band”, and sometimes, it is also incorrectly indicated as sensitivity. A particular case occurs when the sensor operates with a measurand that is in the proximity of zero: in this case, the term “threshold” is preferred to the one resolution and it assumes the meaning of “the minimum value of the measurand which results in a significantly different output with respect to the one obtained when the measured value is null”. In terms of measurements, two key factors are taken into account to characterize a FBG sensor, i.e., the accuracy of the measurement and its bandwidth. The first is the accuracy with which the output power can be measured at the receiver in spite of system noise levels (%). The latter is the bandwidth for which the variations of the examined magnitude can be detected. Besides the measurement bandwidth, other figures of merit of FBGs are the system bandwidth, i.e., the bandwidth that the receiver must have to analyse the system, as well as the sensor stability and dynamic range. The stability is the ability of the sensor to keep its operating characteristics unaltered for a relatively long period of time (e.g., months or years). It is expressed as the maximum variation that can occur in the output, in absolute, relative or reduced value, with same measurand, and operating conditions within a given time interval. Sometimes the term “drift” (offset or shift) is used, with a similar meaning. The dynamic range is the ratio between the maximum and minimum value of the magnitude that can be measured with the required accuracy (dB).

The FBG strain sensors are also classified according to their attenuation spectrum, dispersion spectrum, type of modal propagation, geometric and physical properties of the guiding structure, and to the properties of the mantle. The attenuation spectrum represents the attenuation on the basis of the wavelength in the operation point, while the dispersion spectrum represents the refractive index on the basis of the wavelength in the operation point. The type of modal propagation within the guiding structure (optical fibre or waveguide) depends on the operating principle of the sensor and can be related to the presence of one (unimodal propagation) or multiple modes (multi-modal propagation).

The geometric and physical properties of the guiding structure (optical fibre or waveguide) depend on the materials used, the core and cladding dimensions, the index refraction profile (step index or graded index), the index contrast between core and cladding and the cutting wavelength for operating in unimodal or multimodal regime. The mantle’s properties are represented by the type of mantle (primary or secondary in the case of the optical fibre), breaking resistance, maximum working temperature, and radiation resistance in highly radioactive environments.

## 4. Physics of Fibre Bragg Grating

**Figure 4:**
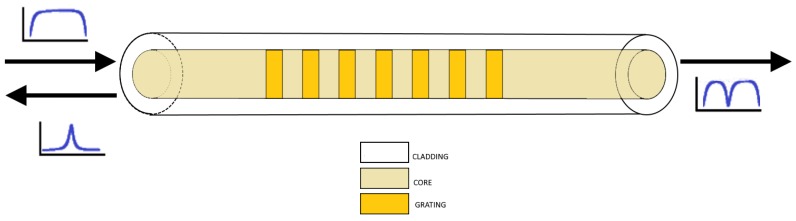
FBG index profile. In insets, the spectrum of the incident, reflected and transmitted beams.

A fibre Bragg grating is an optical device obtained through introducing a modulation of the refractive index of the fibre core. The manufacturing process of FBG (generally called inscription or “writing”) could be realized with interferometric method, direct point-by-point method, and continuous core-scanning method [9,16,17]. The interferometric technique relies on exposing a photo-sensible region of the fibre to an interferometric fringe pattern in order to obtain the whole grating. The pattern is realised through illuminating a proper mask that defines the FBG period. The same results could be obtained with mask-less techniques. Like in the direct point-by-point method, this method is based on the nonlinear absorption of a laser pulse; with this technique, each grating element could be obtained by controlling the laser parameter and the movement of the fibre placed on a translational stage. Similarly, in the continuous core-scanning technique the inscription is realized by controlling only the movement parameters of the translational frame at which the fibre is anchored. There are different kinds of index modulations; for a simple FBG, as in Figure 4, the perturbation is periodic, equally spaced and the modulation strength is only positive. Then, the refractive index along the grating varies as a raised square wave with a duty cycle of half period as shown in the following equation:

(3) $$\left\{ \begin{array}{l} n_{c}=n_{c0}\;0<z<\Lambda /2\\ n_{c}=n_{c0}+\Delta n\;\Lambda /2<z<\Lambda \end{array}\right.$$

where *n_c0_* is the refractive index of the unperturbed core, Δ*n* represents the modulation strength, and Λ is the grating period.

The light propagating in forward direction through the core of an optical fibre is reflected due to the discontinuity at the layer interfaces, while the remaining part passes over. This way, the beam is divided in two components that travel in opposites directions. This phenomenon causes the coupling of the incident wave with the counter-propagating one [18]. At particular wavelengths defined by the grating parameters, the Bragg condition is satisfied; in this case, the contributions of the light reflected at each interface interfere constructively. The resulting backward-propagating spectrum presents a peak centred at the Bragg wavelength while the transmission spectral response presents a notch filter like transfer function. For these reasons is widely used for filtering application or as reflector in communication systems. The light components far from the Bragg wavelength, instead, will pass almost unperturbed. The Bragg wavelength, *λ_B_* is defined by the following the formula:

(4) $$\lambda_{B}=2n_{eff}\Lambda$$

where *n_eff_* is the effective refractive index and Λ the grating pitch.

The reflectivity and transmittivity spectrum could be accurately calculated by using the coupled-mode theory [19]. For a simple FBG, the transfer functions depend on the AC coupling coefficient κ, the DC self-coupling coefficient σ and the length of the grating L defined as follow:

(5) $$\sigma =\frac{2\pi }{\lambda }\bar{\delta n_{eff}}$$

(6) $$\kappa =\frac{\pi }{\lambda }\nu \bar{\delta n_{eff}}$$

(7) L=NΛ

where $\bar{\delta n_{eff}}$ is the DC index change spatially averaged over a grating period, *ν* is the order of the FBG (unitary for a uniform FBG), and N number of periods. Then, the reflected amplitude and power are given by Equations (8) and (9), respectively:

(8) $$\rho =\frac{-\kappa \sinh\left(\sqrt{\kappa^{2}-{\hat{\sigma }}^{2}}L\right)}{{\hat{\sigma }}^{2}\sinh\left(\sqrt{\kappa^{2}-{\hat{\sigma }}^{2}}L\right)+i\left(\sqrt{\kappa^{2}-{\hat{\sigma }}^{2}}\right)\cosh\left(\sqrt{\kappa^{2}-{\hat{\sigma }}^{2}}L\right)}$$

(9) $$r=\left|\rho^{2}\right|=\frac{\sinh^{2}\left(\sqrt{\kappa^{2}-{\hat{\sigma }}^{2}}L\right)}{\cosh^{2}\left(\sqrt{\kappa^{2}-{\hat{\sigma }}^{2}}L\right)-\frac{{\hat{\sigma }}^{2}}{\kappa^{2}}}$$

The bandwidth or full width half maximum (FWHM) is given by:

(10) $$FWHM_{\lambda }=\lambda_{B}S{\left({\left(\frac{\Delta n}{2n_{c0}}\right)}^{2}+{\left(\frac{1}{N}\right)}^{2}\right)}^{1/2}$$

where *S* [20] is a parameter of the grating that is approximately equal to 1 for high reflectivity grating and 0.5 for weak reflectivity gratings.

**Figure 5:**
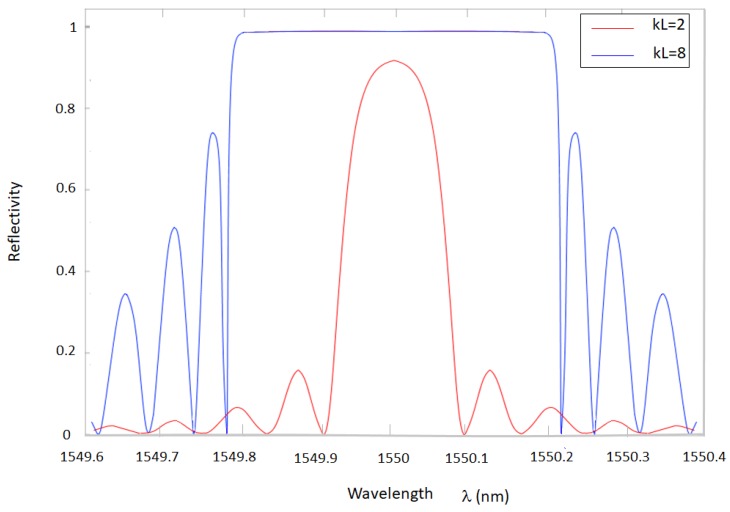
Reflectivity of an FBG with λ_B_ = 1550 nm, kL = 2 (red curve), and kL = 8 (blue curve).

In Figure 5, the reflectivity is plotted with respect to the wavelength for two different values of kL (i.e., 2 and 8) of a uniform FBG. Assuming *n_eff_* = 1.457 and *L =* 1 cm, the bandwidth of the transfer function increases for larger values of the product *kL*, while the drawback is that the side lobe suppression ratio (SLSR) decreases. The length *L* (and the number of periods of length Λ composing the FBG) also affects the bandwidth. Similar results could be obtained increasing $\bar{\delta n_{eff}}$. On the basis of the above considerations, by varying the structural parameters of the grating, such as reflectivity or Bragg wavelength, it is possible to shape the transfer function with high degrees of freedom.

To achieve particular goals, other profiles could be considered. In particular, apodized profile and chirped and phase-shifted profiles are widely used due to their peculiar spectral responses.

The apodization consists in a non-uniform strength of the index modulation along the grating as in the cases of the raised-cosine and the Gaussian profiles.

**Figure 6:**
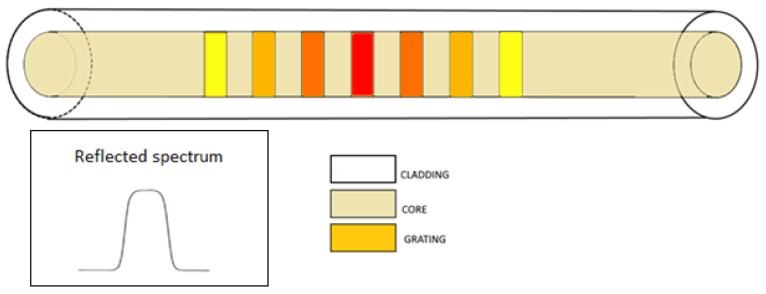
Apodized FBG. Inset: reflected spectrum.

The grating exhibits a lower index modulation strength towards the edges than in the central region; this causes a lower reflection of the wavelengths at the edge of the spectrum than in the uniform case. This way, it is possible to improve the SLRS [21] of the spectrum, as illustrated in the inset of Figure 6.

**Figure 7:**
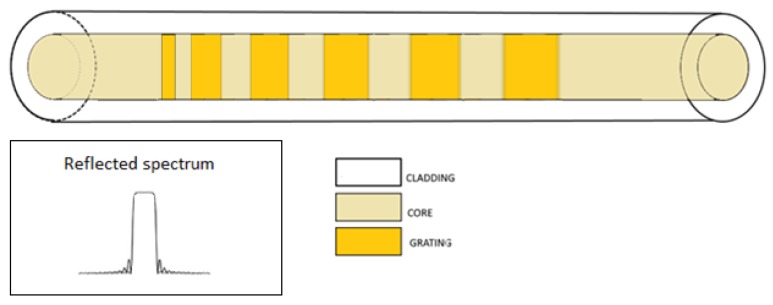
Chirped FBG. Inset: reflected spectrum.

A chirped FBG can be used to increase the bandwidth of the grating as its transfer function presents a region of high reflectivity expanded to a wider range of wavelengths. This can be achieved through modulating the pitch of the grating or through varying the effective refractive index along the direction of propagation (as in Figure 7).

The non-uniform pitch can be considered as a series of several FBGs with slightly different central wavelengths; thus, the total transfer function is modified because of the several wavelengths able to satisfy multiple Bragg conditions [22]. In this case, it is possible to redefine the Bragg wavelength as a function of the axial (z) position, according to the following equation:

(1) $$\lambda_{B}\left(z\right)=2n_{eff}\left(z\right)\Lambda \left(z\right)$$

where both the effective index and the grating pitch are functions of the axial position. For a linearly chirped grating, the grating pitch can be expressed as:

(12) $$\Lambda \left(z\right)=\Lambda_{0}+\alpha z$$

in which α represents the linear proportional coefficient.

**Figure 8:**
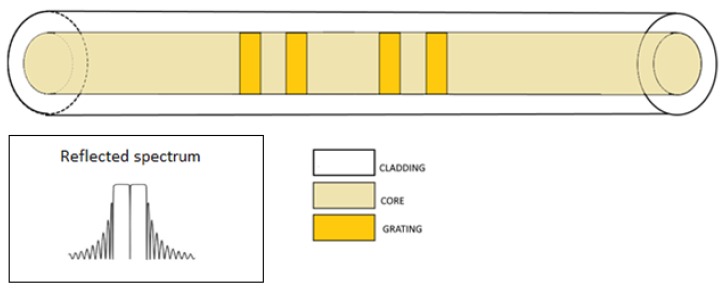
Phase shifted FBG. Inset: reflected spectrum.

In a π-phase shifted FBG (phase shifted FBG PSFBGs), a further π-phase-shift is introduced at the centre of a periodic grating [23]. This configuration consists in two separate FBGs forming a Fabry-Perot (FP), where only a resonance state is allowed; the result of adding a π-phase-shift in the middle of the grating leads to an extremely narrow notch in the reflection spectrum whose position is usually coincident with the Bragg wavelength, as reported in Figure 8. It can also be realized with a detuning phase that differs of π [24]. This way, it is possible to move the notch frequency from the middle of the spectrum.

## 5. Operation Principle of Strain Sensors Based on Fibre Bragg Grating

**Figure 9:**
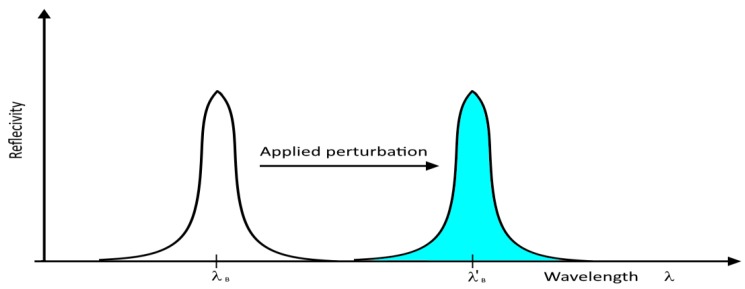
Reflectivity response shift.

According to the previous considerations, the grating properties can be engineered for different applications. The FBGs can be adapted as sensing elements to measure temperature, pressure, strain, vibration, inclination, load, and displacement. A variation in the environmental parameters, for instance, temperature and strain, influence both the pitch and the refractive index of the grating layers perturbing the spectral properties of the FBG, as in Figure 9. Such perturbation in the spectrum is commonly utilized for sensing applications.

By differentiating Equation (4), we can evaluate the shift of the Bragg wavelength due to a uniformly applied strain and temperature changes with the following expression:

(13) $$\Delta \lambda_{B}=2\left[\Lambda \frac{\partial n_{eff}}{\partial l}+n_{eff}\frac{\partial \Lambda }{\partial l}\right]\Delta l+2\left[\Lambda \frac{\partial n_{eff}}{\partial T}+n_{eff}\frac{\partial \Lambda }{\partial T}\right]\Delta T$$

The first term in the above equation contains information about the applied deformation. The shift of the Bragg wavelength due to the strain term *l* is:Δ

(14) $$\Delta \lambda_{B}=2\left[\Lambda \frac{\partial n_{eff}}{\partial l}+n_{eff}\frac{\partial \Lambda }{\partial l}\right]\Delta l$$

where $\frac{\partial n_{eff}}{\partial l}$ is the variation of the effective refractive index induced by the strain and $\frac{\partial \Lambda }{\partial l}$ is the change of pitch. The shift of the Bragg wavelength could be also written as a function of the strain knowing the material properties of the grating as in the following expression:

(15) $$\Delta \lambda_{B}=\lambda_{B}\left[1-\frac{{n_{eff}}^{2}}{2}\left[p_{12}-\nu \left(p_{11}+p_{12}\right)\right]\right]\varepsilon$$

In which *p_ij_* is the Pockel’s coefficient of the stress-optic tensor and ν the Poisson’s ratio.

The applied strain ε can be derived through measuring the λ_B_ shift (i.e., Δλ) according to the relation [25]:

(16) $$\frac{\Delta \lambda_{B}}{\lambda_{B}}=\left(1-p_{e}\right)\cdot \varepsilon$$

where p_e_ is the elasto-optic coefficient that links Δλ/λ with Δ*L/L* through the relation Δλ/λ = *0.79* Δ*L/L* [8]. The second term in the Equation (13) includes the temperature dependence of the Bragg wavelength:

(17) $$\Delta \lambda_{B}=2\left[\Lambda \frac{\partial n_{eff}}{\partial T}+n_{eff}\frac{\partial \Lambda }{\partial T}\right]\Delta T$$

where $\frac{\partial n_{eff}}{\partial T}$ represents the thermo-optic coefficient whose contribution is larger than the one of the term $\frac{\partial \Lambda }{\partial T}$ related to the thermal expansion. As previously stated, both changes in effective index and grating pitch, caused by an external perturbation, contribute to the wavelength shift, but their sensitivities to strain or temperature are significantly different. Typical value for strain or temperature induced Bragg wavelength shift is approximately of 1.2 pm/με and 13.7 pm/°C, respectively. These values underline the necessity of temperature compensation in strain sensing applications as well as the possibility to develop proper readout techniques for simultaneous measurement of strain and temperature.

## 6. Operation Principle of Splitting Mode Strain Sensors Based on Bragg Grating Ring Resonators

In References [26,27,28,29,30], FBG gratings are inserted into ring resonators made of optical fibre connected one end to other to create resonant FBGs strain sensors. These resonant sensor systems are mainly built using FBGs with complex scattering sources (SCs) which couple two counter-propagating modes (traveling wave (TW)) in a ring resonator. This mode coupling generates a spectral response characterized by two asymmetric resonance lines (called ”mode splitting” in the scientific literature and used for many applications, besides the strain sensing [31,32,33,34,35,36,37,38].

**Figure 10:**
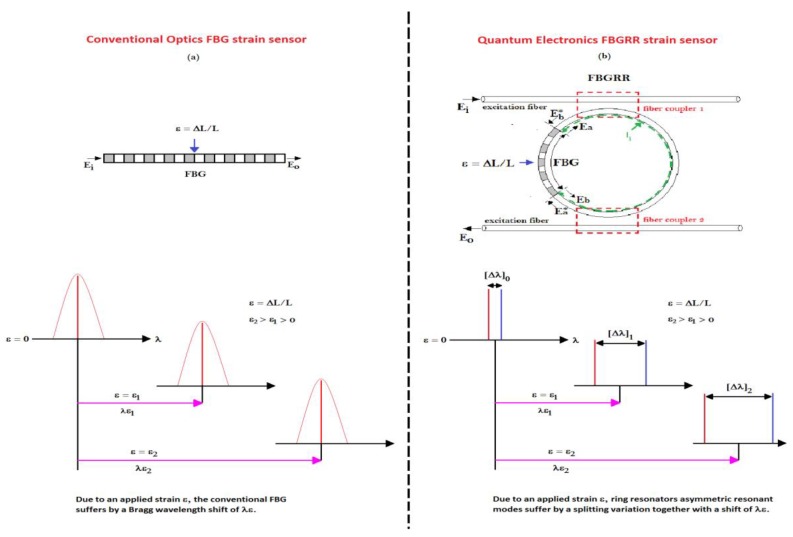
(**a**) FBG conventional static strain sensor; (**b**) fibre Bragg grating ring resonators (FBGRR) strain sensor system, consisting in a FBG inserted into a ring resonator made by an optic fibre closed on itself into a feedback loop, through two fibre couplers.

Each resonance line is created by the combination of two counter-propagating modes. These particular configurations are called fibre Bragg grating ring resonators (FBGRRs) (see Figure 10b). The mode-splitting spectral feature of FBGRRs allows one to measure the strain with a far greater accuracy, making these sensors particularly suited for the structural health-monitoring in civil infrastructures, aerospace, energy, and maritime operations fields. The main advantages of FBGRRs, with respect to the conventional FBG sensors, are represented by a cavity enhanced resolution, which leads to an improved sensing resolution [30], insensitivity to environmental perturbations [26] and lower production costs because, differently from conventional FBG sensors, the sensing scheme is self-referenced and there is no need of a reference signal to perform accurate measurements [26,27,28,29,30].

This technology retains all of the advantages of the fibre-optics, such as compactness, immunity to radio- and electromagnetic interference (RFI, EMI), rapid response for real time monitoring, and ability to be embedded in composite materials in complex structures. Furthermore, the FBGRR based strain sensors can be used in the sensing applications requiring ultra-high resolutions (e.g., seismic monitoring etc.) and insensitivity to the environmental perturbation (e.g., harsh environments typical of industrial or aerospace scenarios).

In Figure 10, two FBG sensor systems are shown: on the left, a conventional FBG static strain sensor based on conventional optics principles (Figure 10a), and on the right, the sensor system consisting of a FBG inserted into a ring resonator made by an optic fibre wrapped on itself into a feedback loop based on mode-splitting principle explained previously (Figure 10b). In a conventional FBG sensor, the strain (ε) corresponds to a change of the FBG length (*L*, equivalent to ε = Δ*L/L*), and (Figure 10a), the information about the measurand is usually encoded by the Bragg wavelength shift of the FBG. In the case of FBGRR, the information about measurand is encoded by the mode splitting of two ”entangled” coupled resonant modes, that are visible as a splitting doublet in the spectral response (see Figure 10b) where the mode splitting is approximately described through two adjacent lines with different colours).

**Figure 11:**
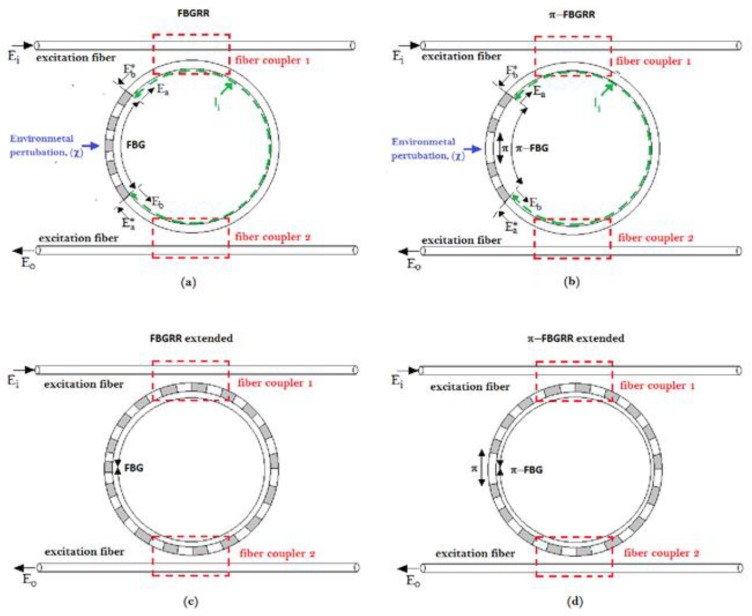
(**a**) FBGRR; (**b**) π-FBGRR; (**c**) extended version of FBGRR; and (**d**) extended version of π-FBGRR.

The following illustrations in Figure 11 shows different architectures based on the mode-splitting operating principle [26,27,28,29,30,31,32,33,34,35,36,37,38,39].

These architectures have been obtained either by introducing SCs through different kinds of fibre Bragg grating inserted in a ring resonator (Figure 11a,b where conventional FBG and π-FBG have been respectively sketched); or by modulating the SC’s lengths through the manipulation of the FBG and π−FBG lengths (Figure 11c,d, representing the extended versions of FBGRR and π-FBGRR, respectively) in order to exploit other physical effects.

The insertion of a π-shifted fibre Bragg grating into a ring resonator (Figure 11b) has been proven to enhance sensing performance [29] for different kinds of environmental parameters/measurands.

With the same architecture sketched in Figure 11b, excluding the experimental record over the static strain sensor resolutions reported in [30], enhanced spectral features of a π-shifted fibre Bragg grating in closed loop have been demonstrated in [36]. In the latter, a performance improvement over a conventional π-shifted fibre Bragg grating has been experimentally proved, also demonstrating that a reduction in sensor’s architecture complexity and manufacturing costs is possible as well.

Being based on mode-splitting principles and the hybridization of technologies already available in the market, Fibre Bragg Grating Ring Resonators represents a new generation of robust, better performing, and cost-effective optical sensors that can be applied to the all the main sectors of strain sensing technologies existing on the market (see Section 9).

## 7. FBG Strain Sensors Interrogation Techniques

**Figure 12:**
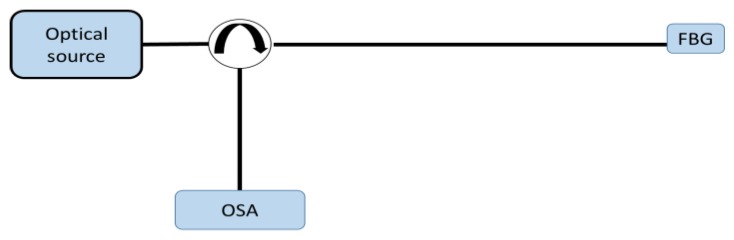
Simple interrogation set up: a broadband optical source is connected to a circulator to feed the FBG, while the FBG reflected beam is redirected to a conventional optical spectrum analyser (OSA) through a circulator.

The interrogation techniques allow one to extract the information about the measurand, encoded in the spectrum produced by the FBG sensors. The spectral change, with reference to the unperturbed condition, is principally associated with a Bragg wavelength shift or with a change in the optical exciting power or in the FWHM. A basic set up that can be used to detect these variations is illustrated in Figure 12, where a broadband optical source is connected to a circulator to feed the FBG. Then, the reflected beam is redirected to a conventional optical spectrum analyser (OSA) connected to the circulator.

Unfortunately, commercial OSAs are costly and slow and, thus, not suitable for dynamic measurements; furthermore they have a typical resolution in the range of tens of picometers while the sensitivity of the FBG strain sensors is of the order of some picometers for one microstrain; thus, to improve the resolution, particular algorithms have to be used [40,41,42].

**Figure 13:**
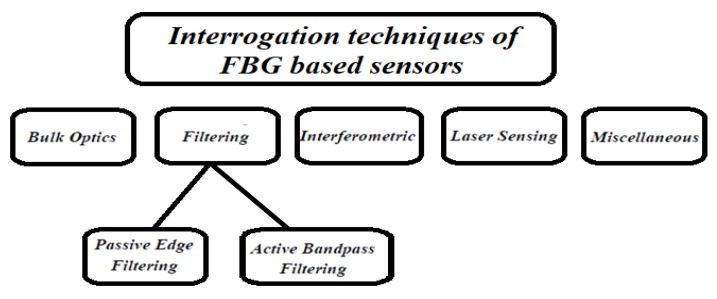
Classification of the interrogation techniques of FBG sensors: bulk optics, filtering, interferometric, laser sensing, and miscellaneous.

For this reason, several demodulation techniques have been conceived and implemented to improve FBG interrogation technique, achieving highest resolutions in terms of spectral detection. According to Reference [9], we can classify the interrogation techniques of FBG sensors into five categories, depending on the main principle adopted in the detecting scheme, as depicted in Figure 13.

The most common interrogation techniques are the filtering and interferometric approaches, in which the read-out of the FBG sensor is done by converting the wavelength shift into a change to an electrical signal. In the following, the review will focus on the implementations of filtering and interferometric techniques. In such settings, the OSA is replaced with a photo-diode receiving the optical signal from the reflected spectrum of the FBG.

**Figure 14:**
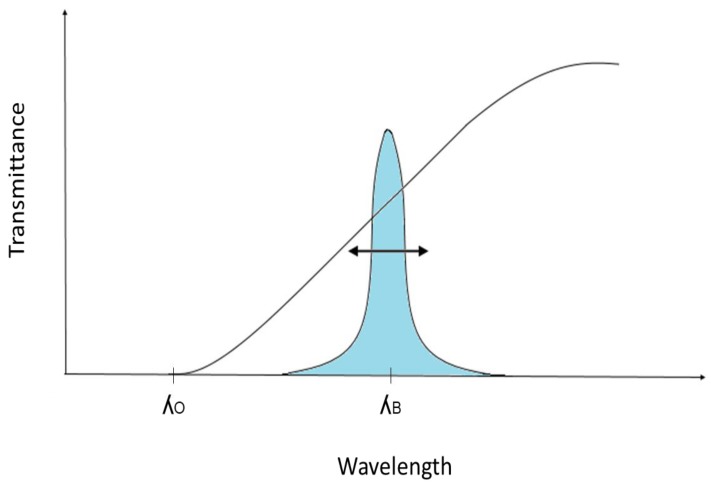
Filter transfer function and FBG spectrum response.

Filtering techniques are divided into passive edge and active bandpass types. In a passive edge filtering, an optical filter presenting a linear attenuation around the wavelength of interest, as shown in Figure 14, is used as the interrogation technique. The change in the FBG spectrum will result in a change on the output power. The correlation between the wavelength and the output power depends on the transmittance function of the passive edge filter.

The optical signal received by the photodiode is proportional to the central wavelength of the input spectrum. The shift of the sensor wavelength produces a variation in the optical power according to the slope of the filter. The main drawback of this methodology is its sensitivity to source power variations [1]. To overcome this limitation, an improved passive edge filtering set-up, where a reference signal was also detected.

**Figure 15:**
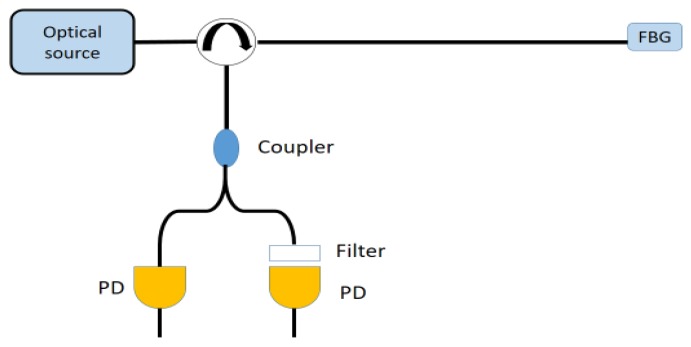
Example of experimental set-up for passive edge filtering interrogation technique with reference signal measurement.

As shown in Figure 15, the input spectrum power at the receiver is divided into two branches by a coupler; the branch for the reference purpose includes only a photodiode (PD) so it receives the unfiltered spectrum power, while the other, which includes filter and photodiode, acts as previously explained. The result of the ratio of the power received from the two photodiodes, as indicated by the Equation (18), is immune to variations in the source and also proportional to the Bragg wavelength shift [1,43].

(18) $$\frac{I_{S}}{I_{R}}=A\left(\lambda_{B}-\lambda_{b}+B\right)$$

where *I_S_* is the filtered intensity of the sensor spectrum, *I_R_* is the unfiltered reference intensity, *A* is a constant determined by the slope of the filter and *B* is a constant arising from the nonzero reflection bandwidth of the FBG. Improvements of intensity based technique are presented in [44,45,46]; in Reference [45] is proposed the use of ultra-short FBGs having shifted matched filters to enhance sensitivity. In Reference [44] is shown a demodulation technique based on a reflective-matched Fibre Bragg Grating scheme to compare the spectrum of the sensing element with its filtered version, while in Reference [46] the reflected optical signal, outgoing from the sensing FBG, pass through two reference FBGs. One of the reference FBGs is matched with the sensing FBG while the second one presents a small offset. The strain could be retrieved after the detection, by subtracting these two signals.

Alternatively, it is possible to take advantage of a large bandwidth light source such as an amplified spontaneous emission (ASE) source (with bandwidth around 20 nm) rather than inserting a filter before the detector. An ASE source presents a power spectrum where the slope of the optical power linearly decreases [47]. The wavelength shift of the Bragg wavelength is directly converted into a variation in the received optical power. This set-up scheme can be simplified into a system consisting of an FBG head sensor with the Bragg wavelength lying in the linear spectral region, and of a photoreceiver.

**Figure 16:**
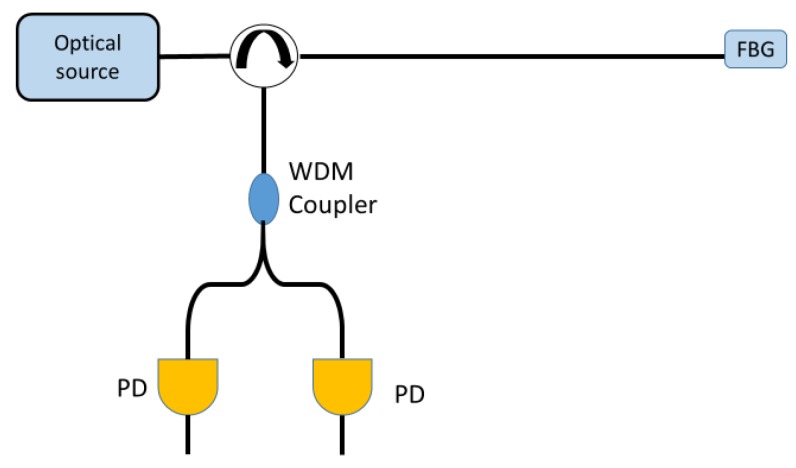
Example of an experimental set-up for passive wavelength division multiplexer coupler filtering interrogation technique.

Another passive filtering interrogation technique involves the use of a wavelength division multiplexer (WDM) coupler as depicted in Figure 16. The WDM is placed at the receiver input and it splits the spectrum power according to the wavelength in two output branches. A photodiode (PD) receiving the optical power is placed in each branch. Since the WDM coupler is a wavelength sensitive element, the power received by two photodiodes change differently when the FBG spectrum shifts due to a perturbation [48].

The ratio between the difference of the power received by each photodiode and the total received power can be related to the change in the Bragg wavelength.

**Figure 17:**
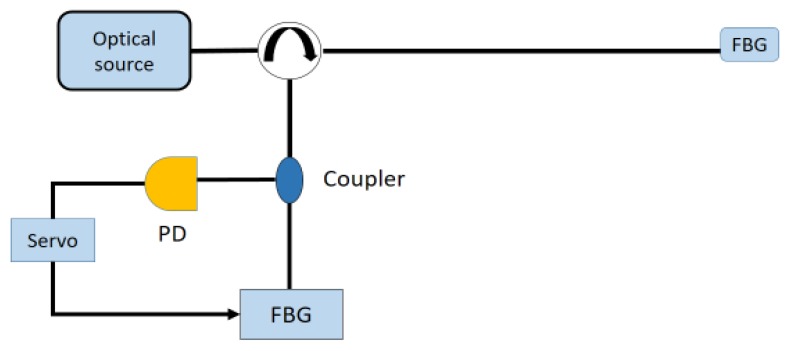
Example of an experimental set-up for active bandpass filtering interrogation technique.

Differently, the interrogation techniques based on the active bandpass filtering rely on the capability of finely tuning a filter. Whereas the FBG sensor is basically a filter, in several configurations the receiver is composed by a matched tuneable filter, like a FBG or a FP equipped with a stretcher. By scanning the spectrum with the stretcher, it is possible to find the peak of reflectivity. The receiver can operate both in scanning or tracking mode. In Figure 17, a possible implementation used to track the Bragg wavelength in a closed loop operation shift is shown. On the basis of the signal coming from the photodiode, the control logic behind the servo scan acts to finely tune the filter through a dither signal on the servo to follow the peak. Through analysing the signal acting on the tuner, it is possible to retrieve the Bragg wavelength shift and to achieve an improved sensitivity [9]. This set-up is also useful in quasi-distributed sensing systems where several different FBGs are placed along the fibre. In order to interrogate each FBG with the same receiver, the scan mode is used to tune on the “colour” of the sensing element (as in the quasi-distributed sensing systems of Figure 2) and to match its Bragg Wavelength. Alternatively, several matched filters can be used to achieve a parallel interrogation set-up where each filter at the receiver stage is a replica of the single sensor configuration.

**Figure 18:**
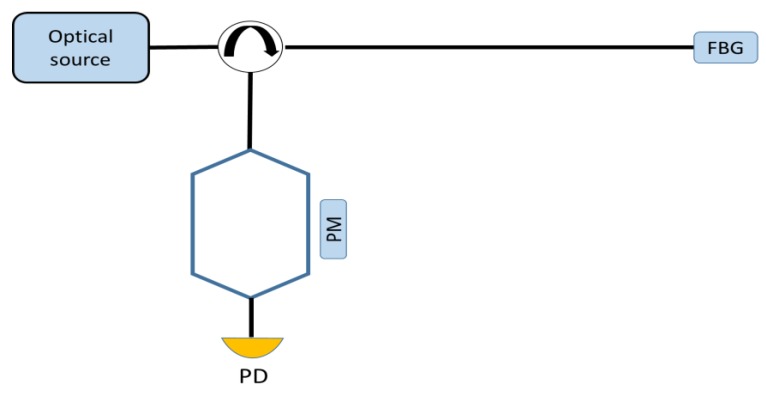
Example of an experimental set-up for interferometric interrogation technique.

Interferometric readout techniques associate a Bragg wavelength shift to an optical phase difference. Often, the receiver includes an unbalanced asymmetric Mach Zehnder interferometer receiving the reflected spectrum of the sensing grating as the input [9], as reported in Figure 18 where a phase modulator (PM) in inserted in one of two arms of the interferometer. Knowing the phase of the interferometer and its intensity output, the change in the measured intensity is transduced into a phase difference. The wavelength shift can be evaluated with a proper signal processing applied on the photodiode voltage (e.g., phase demodulation for interferometric fibre optic sensors). By means of these readout techniques, resolutions in the range of pm/√Hz can be achieved.

The FBG in strain sensing systems is used also to create a Fabry Perot (FP) resonator acting as the sensing element. When a strain is applied to the FP, and composed by two matched FBGs, the resonance wavelength of the FP changes. Different FP-FBG strain sensors adopt the Pound-Drever-Hall (PDH) technique, a laser locking technique that allows improved strain resolutions by suppressing the noise sources associated to the laser intensity fluctuations.

**Figure 19:**
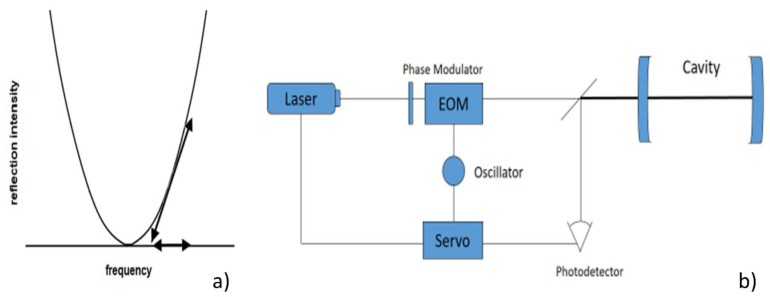
(**a**) Cavity reflection intensity vs. frequency; (**b**) example of an experimental set-up for PDH techniques.

Differently than the active filtering technique in which the receiver is locked to the source, PDH readout techniques lock the laser source to the resonance frequency of FP cavity by means of a feedback loop. The main principle of operation is based on the FP reflection intensity frequency response that reaches a minimum at FP resonance (Figure 19a). Although the reflection coefficients and the corresponding reflected power are symmetric near the resonance wavelength, its derivative changes sign [49]. To recover information about the derivative of this function, the spectrum is swiped near the resonance of the cavity by a phase modulator.

An ideal set-up, as from Figure 19b, consists of a narrow laser source tuned by a servo that operates in a closed loop control feedback. The beam, directed in the FP cavity, is phase-modulated by an electro-optic modulator (EOM), according to the modulating signal. The intensity of the light reflected by the FP cavity is received by a photo-detector. This signal is mixed with the modulating signal to extract the error signal, i.e., the derivative of the reflected signal, after a filtering stage. The servo actuator receives the error signal and closes the loop locking the laser on FP resonance. This laser stabilization technique is convenient in sensing applications since the error signal contains also information about the resonance shift of the FP cavity due to external noise sources.

## 8. Performance Evaluation of the Strain Sensors

This section discusses the state of the art of the strain sensor systems available in literature, focusing on the type of single-point sensing elements, the interrogation techniques, the detection limit, and/or the sensitivity. Two main research approaches are followed in literature; the first is focused on the improvement of performance of the sensing elements, while the second one is focused on the improvement of the read-out systems. To develop new sensing heads, researchers have used different materials or fabrication techniques where the different figures of merit and features of strain sensors are listed, such as sensitivity, detection limit, interrogation technique, punctual optical sensing element and perturbation nature (some examples are summarized in Table 1). In Reference [50] the single-point sensor consists in an all-solid FBG inscribed into an optical fibre using a near-field phase mask and an infrared femtosecond laser. This structure has a cross-section composed of concentric circles with alternating refractive indices. The interrogation technique consists in using a laser source while the FBG was positioned on a mechanical stretching stage. The strain has been measured through the FBG transmission peak shift with a sensitivity of 1.10 pm/με and a detection limit of 26 με. The FBG proposed in Reference [51] is sandwiched between two different expansive materials (one with a positive and one with a negative expansion coefficient). This sensor has shown a sensitivity of 1.5 pm/με if the strain is measured as the Bragg wavelength shift. In another implementation [52], an FBG is realized with a stretched polydimetilsiloxane (PDMS) micro-diffraction grating consisting in a patterned layer of a biocompatible soft polymer, the polydimetilsiloxane (PDMS), micro-fabricated with soft-lithography techniques. The grating has been placed between two glass plates fixed onto two translating stages and the experiments report a resolution of 0.005 ε. In Reference [53], a passive and active strain sensing architecture has been reported. In the passive configuration, the analysis based on the Bragg wavelength shift and full width half maximum change due to the strain variation has shown sensitivities of 104.1 pm/με and 61.6 pm/με, respectively. On the other hand, the FBG filter in a ring laser cavity configuration achieved a resolution of 700 nε and a sensitivity of 74.5 pm/με. The sensor configuration of Reference [54] is based on a FBG pair (with different Bragg wavelengths): one of them is etched while the other shows an un-etched region of polymer fibre (POF). The strain has been applied by using a translation stage in order to measure the sensitivity. The un-etched FBG sensitivity is 1.24 pm/με, while the etched FBG one is 1.65 pm/με. Careful calibration of the characterization matrix coefficients can allow measurements of temperature and strain simultaneously.

The strain sensitivity of these sensors can be improved using an etched and regenerated fibre Bragg grating [55]. The measurements have reported a reflectivity of 69.8% and a linear strain sensitivity of 4.5 pm/με, stable on a large temperature range. The authors in Reference [56] succeeded to realize a dual-FBG temperature compensated strain sensor using a stable wavelength tunable inscription of polymer fibre. The Bragg wavelength (λ_B_) of the sensor can be tuned over 7 nm with the inscription with a 325 nm HeCd CW laser. Through using one FBG as the reference and the other as the sensing element, the system achieved a sensitivity of 0.73 pm/με.

We summarized the interrogation methods and their important features in Table 2. The experiment reported in Reference [57] is focused on the improving the read-out through a specific spectroscopic technique. In the proposed method, the FBG is mounted onto a piezo (PZT) translation stage stretching the punctual element and the source is modulated at a radio-frequency. The readout based on high-frequency mixer demodulation was shown to be sensitive to the perturbation of FBG. This system achieves a strain resolution below the με and a sensitivity of 0.94 pm/με.

In Reference [58], the stretched FBG output is post-processed using a degenerated four wave mixing (FWM) for frequency chirp magnification to enhance the strain sensitivity. The proposed system was shown to reach strain and temperature sensitivity of 5.36 pm/με and 54.09 pm/°C, respectively.

Another technique [59] shows the use of the comb spectroscopy. The read out system relies on two comb phase locked lasers whose outputs pass through different fibre paths, one of them including a sensitive FBG. At the end of the fibre paths, the fields are optically summed and analysed. This system operates statically up to 1THz with a sensitivity of 34 nε.

In Reference [60], the authors describe a measurement system based on a Fabry-Perrot cavity made of two FBGs. The setup is realized with two arms (reference and sensing), each one containing: a modulator, a Fabry-Perot interferometer (FFPI), a photodiode and an analysing system. The laser interrogates the first FFPI with a phase modulator whereas the second FFPI is interrogated with sidebands using an intensity modulator. The reflected light from the FFPIs is demodulated and, by using a cross-correlation algorithm, the resonance difference between the two FFPIs is evaluated. A strain resolution of 0.63 nε was reported.

The interrogation system implemented in Reference [23] uses a π-phase-shifted FBG as a sensing element. Through a laser tuning technique based on driving the voltage with a triangle wave shape, the authors improved the wavelength scanning stability of the tuneable laser. A strain resolution of 0.83 nε in vacuum was achieved by using a static strain demodulation algorithm based on Gauss curve fitting and peak detection.

In Reference [61], the authors proposed a sensor realized with two FFPIs, one for sensing and the other one acting as reference. Differently from Reference [43], a Pound-Drever-Hall technique is employed as the interrogation technique in the readout. Furthermore, a cross-correlation algorithm is used to measure the strain with a resolution of 4.5 nε. The dynamic range can reach to hundreds of με, and the measuring period is a few tens of seconds.

The configuration implemented in Reference [62] enables the simultaneous interrogation of an array of FBGs with a wavelength-swept fibre laser, for static and dynamic strain measurements. The sensitivity is 1 με in static, while the dynamic sensitivity was reported as 3.4 nε/√Hz at 500 Hz.

In Reference [63], the authors proposed a system made by a mechanically and acoustically isolated FFPI onto PZT stretcher. The strain signals are encoded with a laser phase-locked to a H^13^C^14^N gas cell. The readout is carried out by an FPGA using the H^13^C^14^N absorption line. The system was reported to reach to a resolution less than 1 nε at 20 MHz.

Another solution based on the beating signals is explained in Reference [64]. The authors developed a dual-frequency optoelectronic oscillator (OEO) as an interrogation system incorporating two phases shifted FBGs (PSFBGs) one of which used as reference while the other is the strain sensing element. The two PSFBGs, together with two laser diodes, a phase modulator and a photodiode, implement two microwave photonic filters (MPF), each one having two pass-bands. This system generates two microwave signals for which the beating is sensitive only to the strain. A sensing resolution of 0.83 με and a sensitivity of 119.2 MHz/με was demonstrated.

In Reference [65], the authors used an optical attenuator to improve the sensitivity of an FBG sensor. A slight enhancement was obtained by carefully controlling the optical attenuation. The sensitivity to the strain and temperature was improved through optimizing the value of the amplification factor. The strain sensitivity is 2.40 pm/ με with an optical attenuation of 3 dB.

The Bragg frequency difference could be also retrieved in birefringence-introduced in which the frequency difference act as discernment of strain and temperature through using a π-phase-shifted FBG in PANDA-type fibre [66]. A double-sideband interrogation method has been implemented as a readout technique, and a resolution of 0.018 με has been obtained in the experiment of simultaneous measurement of strain and temperature.

A different configuration based on OEO has been reported in Reference [67]. In particular, a frequency-switchable OEO is used as interrogation system for a PS-FBG, inscribed in a few-mode fibre. In this system, the two microwave signals generated by the OEO, exhibit different strain and temperature sensitivities of 54.3 MHz/με and 58.5 MHz/με respectively.

To achieve a temperature compensated strain measurement, the authors in Reference [68] use two matched pairs of chirped FBGs. For each pair of chirped FBGs, an FBG is placed in the sensing area, so that an FBG is dedicated to measure temperature while the other is placed on a cantilever for the strain sensing. The proposed intensity interrogation system demonstrates a strain sensitivity of 750 pW/ με and a detection limit of 1 με.

Being the FBG a phase sensitive element, slow light enhances a phase change due to external perturbations: indeed, the sensor reported in Reference [69] uses the slow light principle to enhance sensitivity. This enhancement is proportionally to the group index, or, in other words, to the reciprocal of the group velocity. By using a slow-light configuration of FBG, with a group index of 127, a strain sensor with a detection limit of 880 fε/√Hz in the 20-kHz range and a sensitivity of 3.14 × 10^5^ ε^−1^ has been obtained. The same group reported [70] further improvements in FBG strain sensor using slow light in a low loss FBG. Experimental tests have proven that this configuration is capable of detecting strains with a detection limit of 280 fε/√Hz in the 20-kHz range and with a sensitivity of 2.1 × 10^6^ ε^−1^. The authors of [8] reported strain measurements with a record detection limit in the range of 10^−13^ ε/√Hz. This result has been experimentally recorded with a fibre Bragg-grating resonator locked to a Pound-Drever-Hall stabilized source, while the active interrogation of the sensor has been performed through a secondary carrier. In Reference [26], the authors described the experimental demonstration of an optical ring resonator, including a FBG. By exciting the system far from the FBG reflectivity peak, the system acts as a common ring resonator, while a split-mode structure appears near Bragg wavelength. This FBG-RR enhances local strain measurements and it is almost insensitive to the environmental changes. In Reference [30], a π-shifted-FBG ring cavity is used as strain sensor and it is interrogated with a method exploiting the physics of the mode splitting, as in Reference [18]. The mode splitting spectral response makes this device not only extremely sensitive to environment changes, but also to the grating refractive index. The interrogation with a locked-carrier scanning-sideband technique is performed demonstrating a detection limit of 320 pε/√Hz. Another interrogation technique is reported in Reference [71], where the polarization-spectroscopy interrogation technique has been used for a fibre Bragg-grating Fabry-Pérot cavity, acting as sensing element. The PDH technique has been implemented and the frequency discrimination has been generated exploiting the fibre birefringence. This method demonstrates static and dynamic mechanical sensing below the pico-strain level.

## 9. Key Sectors and Main Market Players Composing the Global FBG Strain Sensors Market

In this section we report several FBG strain sensor applications at research stage with a potential industrial implication, as well as the key sectors in the global fibre-optic strain sensors market.

Several research groups have been involved in developing new systems to retrieve information from the spectrum of FBG sensors as summarized in Table 3, where the sensitivity, the detection limit, the interrogation technique, the punctual optical sensing element and the kind of perturbation investigated are reported.

A sensor for a long-distance remote system (50 km) is described in Reference [72], where the residual Raman pump output laser (after the SMF) excites the ASE of an EDF. The light beam coming from ASE is passed through the FBG and a dip in the reflected spectrum becomes visible. By measuring the position of the spectral dip, the sensor can correlate the spectral information to the strain. The system achieved a strain sensitivity of 1.1 pm/με with a root mean square error of 9.5 pm.

The FBG based strain sensing systems have been also used in the crustal deformation applications [73]. For the measurement, a pre-strained π-FBG was mounted between two piers anchored to the rock, while a fibre ring was placed close to the π-FBG, strain isolated. The crustal deformation was obtained through keeping the fibre ring and π-FBG at the same temperature and comparing their resonance frequencies. A minimum strain of 10 nε was measured by using a narrow line-width tuneable laser (line-width of 100 kHz).

The fibre optic strain gauge system can be used in structural monitoring and smart-structure applications [48]. The strain gauge based on FBG and wavelength demodulation can measure the wavelength of the narrow-band back-reflected spectrum from the grating at the sensing head. Both the static and dynamic strain can be measured with a shot noise-limited resolution of 0.44 με/√Hz, a 27.8 dB of dynamic range and a bandwidth of 250 Hz.

A different configuration for crustal movement sensor was realized using two couples of FBGs, with each couple incorporated into an optic fibre path [74]. One path is used as reference while the other is stretched by a PZT stage. The tuneable laser output is split into the paths with a Y-branch and these beams are passed through the FBG pairs. The reflected spectrum is analysed by varying the strain. The reported resolution of the system is 2.6 nε. A harsh environment experiment was carried out to measure the strain on a low temperature superconducting magnet [75]. Four FBGs were attached onto the magnet, but the characterization of the FBGs was carried out by putting it onto a cantilever. The system reached to a sensitivity of 1.4 pm/με.

We report the key sectors composing the global fibre-optic strain sensors market with reference to the previous strain sensing technologies in Table 4. The list is not exhaustive and includes only the key industry sectors with the largest use of fibre-optic strain sensors today. The number of sectors in the list is expected to grow in the coming years, due to the increasing availability and diminishing cost of the technology, potentially allowing penetrations in other market sectors. Moreover, we report the main market players of the fibre optics strain sensing technology in Table 5.

## 10. Conclusions

In this review, fibre Bragg grating strain sensing technologies and their applications have been reported. The underlying physical principles, interrogation/readout techniques, and the main parameters for evaluating the performance of fibre Bragg strain sensors have been reviewed. Furthermore, recent advances towards highly-sensitive FBG heads and their specific applications have been extensively discussed. We also reviewed novel configurations including Bragg grating strain sensors based on mode splitting. The principle of operation, performance, and benefits of FBG strain sensors are highlighted and compared to give a perspective on the state of the art. Finally, the key market sectors and the main market players, composing the global fibre-optic strain sensors market, are also investigated.

## Figures and Tables

**Table 1:** Figures of merit and features of some examples of static strain sensors.

Reference	Sensitivity	Detection Limit	Interrogation Technique	Punctual Optical Sensing Element	Perturbation
[50]	1.10 pm/με	26 µε	Laser, and mechanical stretching	All solid FBG	Static
[51]	1.5 pm/με		Laser, optical analyser	FBG embedded with expansive material	Static
[52]		0.005	Laser	Stretched polydimetilsiloxane (PDMS) micro-diffraction grating	Static
[53]	Wavelength shift and FWHM of 104.1 pm/με and 61.6 pm/με respectively	700 nε (active)	A broadband optical source OSA to perform readings	3 mm long FBG written in photosensitive SMF	
[54]	Un-etched fibre Bragg grating 1.24 pm/με, Etched FBG 1.65 pm/με		Spectroscopic techniques OSA	Bragg grating pair, with one grating in the etched and the other in un-etched region of polymer fibre strain applied using a translation stage setup	Static
[55]	4.5 pm/με		Wavelength shift Through OSA	etched and regenerated FBG onto stretcher	Static
[56]	0.73 pm/με		Laser, reflectivity spectrum shift	dual-FBG strain sensor in PMMA single-mode microstructured POF	Static

**Table 2:** Interrogation/read-out technique.

Reference	Interrogation/Read-Out Technique	Features
[26]	Detected by a photodiode and visualized on a digital oscilloscope to measure splitting	resolution improvement
[30]	PDH to lock the laser to the FBG to measure splitting	resolution improvement
[57]	Radio-frequency modulation spectroscopic techniques	
[58]	Laser with post-processing sensitivity enhancement using FWM	sensitivity enhancement
[59]	Dual-comb spectroscopy	resolution improvement large dynamic range
[60]	Sideband interrogation technique (~2 MHz rf signals) to measure the resonance difference between a pair of FFPI	resolution improvement
[23]	Triangle wave driver Laser voltage, demodulation algorithm based on Gauss curve fitting and peak detection	resolution improvement
[61]	PDH technique	resolution improvement large dynamic range
[62]	Wavelength-swept fibre laser into unidirectional ring	simultaneous interrogation of quasi distributed sensor
[63]	Laser encoding Strain Signals. Read out using a H^13^C^14^N absorption line	resolution improvement
[64]	Dual-frequency OEO, ESA	thermal-insensitivity
[65]	Weak value amplification based on optical attenuation	sensitivity improvement
[66]	Double-sideband interrogation scheme	simultaneous measurement of strain and temperature
[67]	Radio-frequency modulation spectroscopic techniques	simultaneous measurement of strain and temperature
[68]	Change in voltage measured by Photodiode1.	temperature compensation
[69]	Tunable laser source controlled by the FBG modulated transmitted power	resolution improvement
[70]	Tunable laser source controlled by the FBG modulated transmitted power	resolution improvement
[8]	PDH to lock the laser to the FBG	resolution improvement
[71]	PDH to lock the laser to the FBG	resolution improvement

**Table 3:** Applications at research stage.

Reference	Sensitivity	Detection Limit	Interrogation Technique	Punctual Optical Sensing Element	Perturbation
[72]	1.1 pm/με	RMS error 9.5 pm	Spectrum ASE generated by EDF and passed through the FBG	FBG onto tensile stage system	static
[73]		10 nε	Narrow line-width tuneable laser line-width 100 kHz	FBG onto two piers anchored to the rock and free FBG as reference	static
[48]		0.44 μϵ/√Hz	Resonant wavelength of the back-reflected spectrum from the grating	FBG strain gauge	quasi-static
[74]		2.6 nε	Tuneable laser	Pairs of equal FBG into two branches, one used as reference	static
[75]	1.4 pm/με		Laser	FBG onto cantilever	static

**Table 4:** Key sectors in the global fibre-optic strain sensors market.

**Oil & Gas**	**Energy**	**Military**
Pipelines [76,77,78] Seismic [79,80] In-well [81]	Wind turbines [82] Geothermal [83] Smart Grids [84]	Hydrophones [85,86] Shipboard [87]
**Industrial**	**Civil**	**Homeland Security**
Process control [88] Machine tools [89]	Landslides [90]	Intrusion detection [91,92] Chemical detection [93]
**Biomedical**	**Aerospace**	**Transportation**
Heart rate Respiratory rate [94] Respiratory movement [95] MRI [94,95]	Fuselages [96,97]	Rail monitoring [98,99]

**Table 5:** The main market players.

3M Optical OEM Systems	[100]
Advanced Optical Solutions GmbH	[101]
AFW Technologies Pty Ltd.	[102]
Alcatel Optronics	[103]
Alxenses Company Ltd.	[104]
Ascentta Inc.	[105]
AtGrating (China)	[106]
Broptics Technology Inc.	[107]
DK Photonics Technology Co., Ltd.	[108]
FBG TECH	[109]
FBGS International NV	[110]
Fibreguide Industries	[111]
Gould Fibre Optic	[112]
HBM FibreSensing, S.A.	[113]
InPhoTech	[114]
ITF Technologies Inc.	[115]
iXBlue	[116]
Keysight	[117]
MPB Communications	[118]
Optoadvance S.r.l.	[119]
Optromix Co.	[120]
Oxford Lasers Inc	[121]
OZ Optics Limited	[122]
Proximion AB	[123]
Technica Optical Components, LLC	[124]
TeraXion	[125]
Thorlabs Inc.	[126]
VFIBRE Ltd.	[127]

## References

[1] Yu F.T.S., Yin S. (2002). Fiber Optic Sensors.

[2] Yin S., Ruffin P., Yu F. (2008). Fiber Optic Sensors.

[3] Gupta B.D. (2006). Fiber Optic Sensors.

[4] Udd E., Spillman W. (2011). Fiber Optic Sensors: An Introduction for Engineers and Scientists.

[5] Marcus M.A. (2005). Fiber Optic Sensor Technology and Applications IV. Proceedings SPIE Conference.

[6] Lubliner J. (2008). Plasticity Theory (Dover Books on Engineering).

[7] Rees D. (2006). Basic Engineering Plasticity: An Introduction with Engineering and Manufacturing Applications.

[8] Gagliardi G., Salza M., Avino S., Ferraro P., Natale P.D. (2010). Probing the Ultimate Limit of Fiber-Optic Strain Sensing. Science.

[9] Albert A.C.A.C.J. (2013). Fiber Bragg Grating Sensors: Recent Advancements, Industrial Applications and Market Exploitation.

[10] Thévenaz L. (2006). Review and Progress on Distributed Fibre Sensing. Opt. Fiber Sens..

[11] Campanella C.E., Ai G., Ukil A. Distributed fiber optics techniques for gas network monitoring. Proceedings of the 2016 IEEE International Conference on Industrial Technology (ICIT).

[12] Cieszczyk S., Kisaa P. (2016). Inverse problem of determining periodic surface profile oscillation defects of steel materials with a fiber Bragg grating sensor. Appl. Opt..

[13] Barrias A., Casas J., Villalba S. (2016). A Review of Distributed Optical Fiber Sensors for Civil Engineering Applications. Sensors.

[14] Li X., Zhang Z., Li L. (2017). Wind direction sensing system based on fiber Bragg grating sensor. Appl. Opt..

[15] Zheng S., Zhang N., Xia Y., Wang H. (2014). Research on non-uniform strain profile reconstruction along fiber Bragg grating via genetic programming algorithm and interrelated experimental verification. Opt. Commun..

[16] Mihailov S.J. (2012). Fiber Bragg Grating Sensors for Harsh Environments. Sensors.

[17] Joe H.E., Yun H., Jo S.H., Jun M.B., Min B.K. (2018). A review on optical fiber sensors for environmental monitoring. Int. J. Precis. Eng. Manuf. Green Technol..

[18] Chuang S.L. (2009). Physics of Photonic Devices.

[19] Pierce J.R. (1954). Coupling of Modes of Propagation. J. Appl. Phys..

[20] Russell P.S.J., Archambault J.L., Reekie L. (1993). Fibre gratings. Phys. World.

[21] Khan S.S.A., Islam M.S. (2012). Determination of the Best Apodization Function and Grating Length of Linearly Chirped Fiber Bragg Grating for Dispersion Compensation. J. Commun..

[22] Khare A., Singh J. (2011). Design and Study of Chirped Fiber Bragg Grating for Sensing of Hazardous Gases. Int. J. Comput. Appl..

[23] Huang W., Zhang W., Zhen T., Bian C., Du Y., Li F., López-Higuera J.M., Jones J.D.C., López-Amo M., Santos J.L. (2014). π-phase-shifted FBG for improving static-strain measurement resolution based on triangle-wave laser tuning technique. Proceedings of the 23rd International Conference on Optical Fibre Sensors.

[24] Liu Y., Lee S.B., Choi S.S. (1998). Phase-Shifted Fiber Bragg Grating Transmission Filters Based on the Fabry-Perot Effect. J. Opt. Soc. Korea.

[25] Hill K., Meltz G. (1997). Fiber Bragg grating technology fundamentals and overview. J. Lightw. Technol..

[26] Campanella C.E., Giorgini A., Avino S., Malara P., Zullo R., Gagliardi G., Natale P.D. (2013). Localized strain sensing with fiber Bragg-grating ring cavities. Opt. Express.

[27] Campanella C. (2016). Coupled π-shifted fibre Bragg grating ring resonant strain sensors. Electron. Lett..

[28] Malara P., Campanella C.E., Giorgini A., Avino S., Gagliardi G. (2016). Fiber Bragg grating laser sensor with direct radio-frequency readout. Opt. Lett..

[29] Campanella C.E., Mastronardi L., De Leonardis F., Malara P., Gagliardi G., Passaro V.M.N. (2014). Investigation of fiber Bragg grating based mode-splitting resonant sensors. Opt. Express.

[30] Malara P., Mastronardi L., Campanella C.E., Giorgini A., Avino S., Passaro V.M.N., Gagliardi G. (2014). Split-mode fiber Bragg grating sensor for high-resolution static strain measurements. Opt. Lett..

[31] De Leonardis F., Campanella C., Troia B., Perri A., Passaro V. (2014). Performance of SOI Bragg Grating Ring Resonator for Nonlinear Sensing Applications. Sensors.

[32] Campanella C.M., Dunai M., Calabrese L., Campanella C.E. (2016). Design guidelines for nanoparticle chemical sensors based on mode-splitting silicon-on-insulator planar microcavities. J. Opt. Soc. Am. B.

[33] Malara P., Campanella C.E., Giorgini A., Avino S., Natale P.D., Gagliardi G. (2016). Super-Resonant Intracavity Coherent Absorption. Sci. Rep..

[34] Campanella C.E., Malara P., Campanella C.M., Giove F., Dunai M., Passaro V.M.N., Gagliardi G. (2016). Mode-splitting cloning in birefringent fiber Bragg grating ring resonators. Opt. Lett..

[35] Campanella C.E., De Leonardis F., Passaro V.M.N. (2015). Fiber Bragg grating ring resonators under rotation for angular velocity sensing. Appl. Opt..

[36] Campanella C.E., De Leonardis F., Mastronardi L., Malara P., Gagliardi G., Passaro V.M.N. (2015). Investigation of refractive index sensing based on Fano resonance in fiber Bragg grating ring resonators. Opt. Express.

[37] Malara P., Campanella C.E., De Leonardis F., Giorgini A., Avino S., Passaro V.M.N., Gagliardi G. (2015). Enhanced spectral response of -phase shifted fiber Bragg gratings in closed-loop configuration. Opt. Lett..

[38] Campanella C.E., Campanella C.M., De Leonardis F., Passaro V.M.N. (2014). A high efficiency label-free photonic biosensor based on vertically stacked ring resonators. Eur. Phys. J. Spec. Top..

[39] Campanella C.E., Carlo M.D., Cuccovillo A., Passaro V.M.N. (2018). Loss-induced control of light propagation direction in passive linear coupled optical cavities. Photonics Res..

[40] Tosi D. (2017). Review and Analysis of Peak Tracking Techniques for Fiber Bragg Grating Sensors. Sensors.

[41] Lamberti A., Vanlanduit S., Pauw B.D., Berghmans F. (2014). A novel fast phase correlation algorithm for peak wavelength detection of fiber Bragg grating sensors. Opt. Express.

[42] Tosi D. (2015). KLT-Based Algorithm for Sub-Picometer Accurate FBG Tracking With Coarse Wavelength Sampling. IEEE Photonics Technol. Lett..

[43] Melle S., Liu K., Measures R. (1992). A passive wavelength demodulation system for guided-wave Bragg grating sensors. IEEE Photonics Technol. Lett..

[44] Cui J., Hu Y., Feng K., Li J., Tan J. (2015). FBG Interrogation Method with High Resolution and Response Speed Based on a Reflective-Matched FBG Scheme. Sensors.

[45] Cheng R., Xia L., Ran Y., Rohollahnejad J., Zhou J., Wen Y. (2015). Interrogation of Ultrashort Bragg Grating Sensors Using Shifted Optical Gaussian Filters. IEEE Photonics Technol. Lett..

[46] Zhang Z., Yan L., Pan W., Luo B., Wang P., Guo L., Zhou W. (2012). Sensitivity Enhancement of Strain Sensing Utilizing a Differential Pair of Fiber Bragg Gratings. Sensors.

[47] Kang S.C. Real-time measurement for static and dynamic strain using a fiber Bragg grating and the ASE profile of EDFA. Proceedings of the 13th International Conference on Optical Fiber Sensors.

[48] Melle S.M., Liu K., Measures R.M. (1993). Practical fiber-optic Bragg grating strain gauge system. Appl. Opt..

[49] Black E.D. (2001). An introduction to PoundDreverHall laser frequency stabilization. Am. J. Phys..

[50] Liu N., Li Y., Wang Y., Wang H., Liang W., Lu P. (2011). Bending insensitive sensors for strain and temperature measurements with Bragg gratings in Bragg fibers. Opt. Express.

[51] Li J., Du Y., Liu C. FBG Strain Sensor Based on Thermal Stress Mechnism. Proceedings of the 2008 Second International Symposium on Intelligent Information Technology Application.

[52] Moscato M., Schena E., Saccomandi P., Francomano M., Accoto D., Guglielmelli E., Silvestri S. A micromachined intensity-modulated fiber optic sensor for strain measurements: Working principle and static calibration. Proceedings of the 2012 Annual International Conference of the IEEE Engineering in Medicine and Biology Society.

[53] Ferreira M., Bierlich J., Becker M., Schuster K., Santos J., Frazão O. (2014). Ultra-High Sensitive Strain Sensor Based on Post-Processed Optical Fiber Bragg Grating. Fibers.

[54] Bhowmik K., Peng G.D., Luo Y., Ambikairajah E., Lovric V., Walsh W.R., Rajan G. (2016). High Intrinsic Sensitivity Etched Polymer Fiber Bragg Grating Pair for Simultaneous Strain and Temperature Measurements. IEEE Sens. J..

[55] Wang Y., Qiao X., Yang H., Su D., Li L., Guo T. (2014). Sensitivity-Improved Strain Sensor over a Large Range of Temperatures Using an Etched and Regenerated Fiber Bragg Grating. Sensors.

[56] Yuan W., Stefani A., Bang O. (2012). Tunable Polymer Fiber Bragg Grating (FBG) Inscription: Fabrication of Dual-FBG Temperature Compensated Polymer Optical Fiber Strain Sensors. IEEE Photonics Technol. Lett..

[57] Gagliardi G., Salza M., Ferraro P., Natale P.D. (2005). Fiber Bragg-grating strain sensor interrogation using laser radio-frequency modulation. Opt. Express.

[58] Du J., He Z. (2013). Sensitivity enhanced strain and temperature measurements based on FBG and frequency chirp magnification. Opt. Express.

[59] Kuse N., Ozawa A., Kobayashi Y. (2013). Static FBG strain sensor with high resolution and large dynamic range by dual-comb spectroscopy. Opt. Express.

[60] Liu Q., Tokunaga T., He Z. (2012). Sub-nano resolution fiber-optic static strain sensor using a sideband interrogation technique. Opt. Lett..

[61] Liu Q., He Z., Tokunaga T., Hotate K. An ultra-high-resolution large-dynamic-range fiber optic static strain sensor using Pound-Drever-Hall technique. Proceedings of the 2011 International Quantum Electronics Conference (IQEC) and Conference on Lasers and Electro-Optics (CLEO) Pacific Rim Incorporating the Australasian Conference on Optics, Lasers and Spectroscopy and the Australian Conference on Optical Fibre Technology.

[62] Wang Y., Cui Y., Yun B. (2006). A fiber Bragg grating sensor system for simultaneously static and dynamic measurements with a wavelength-swept fiber laser. IEEE Photonics Technol. Lett..

[63] Lam T.T., Chow J.H., Shaddock D.A., Gray M.B., McClelland D.E. Fiber optic strain sensing using an absolute frequency reference. Proceedings of the 35th Australian Conference on Optical Fibre Technology.

[64] Xu O., Zhang J., Deng H., Yao J. (2017). Dual-frequency Optoelectronic Oscillator for Thermal-Insensitive Interrogation of a FBG Strain Sensor. IEEE Photonics Technol. Lett..

[65] Yoo K.W., Han Y.G. (2017). Sensitivity improvement in fiber Bragg grating sensors using all-fiber weak value amplification based on optical attenuation. J. Lightw. Technol..

[66] Chen J., Liu Q., He Z. (2017). High-Resolution Simultaneous Measurement of Strain and Temperature Using pi-Phase-Shifted FBG in Polarization Maintaining Fiber. J. Lightw. Technol..

[67] Liu J., Wang M., Tang Y., Yang Y., Wu Y., Jin W., Jian S. (2017). Switchable Optoelectronic Oscillator Using an FM-PS-FBG for Strain and Temperature Sensing. IEEE Photonics Technol. Lett..

[68] Maheshwari M., Tjin S.C., Yang Y., Asundi A. (2017). Wavelength-shifted chirped FBGs for temperature compensated strain measurement. Sens. Actuators A.

[69] Wen H., Terrel M., Fan S., Digonnet M. (2012). Sensing With Slow Light in Fiber Bragg Gratings. IEEE Sens. J..

[70] Wen H., Skolianos G., Fan S., Bernier M., Vallee R., Digonnet M.J.F. (2013). Slow-Light Fiber-Bragg-Grating Strain Sensor With a 280 femtostrain/*Hz* Resolution. J. Lightw. Technol..

[71] Gagliardi G., Nicola S.D., Ferraro P., Natale P.D. (2007). Interrogation of fiber Bragg-grating resonators by polarization-spectroscopy laser-frequency locking. Opt. Express.

[72] Lee J., Han Y.G., Chang Y., Lee S. (2004). Raman amplifier based long-distance, remote FBG strain sensor with EDF broadband source recycling residual Raman pump. Electron. Lett..

[73] He Z., Liu Q., Tokunaga T. (2013). Ultrahigh resolution fiber-optic quasi-static strain sensors for geophysical research. Photonic Sens..

[74] He Z., Liu Q., Tokunaga T. Development of nano-strain-resolution fiber optic static strain sensor for crustal deformation monitoring. Proceedings of the 2012 Asia Communications and Photonics Conference (ACP).

[75] Zhang H., Wang Q., Wang H., Song S., Zhao B., Dai Y., Huang G., Jiang Z. (2010). Fiber Bragg Grating Sensor for Strain Sensing in Low Temperature Superconducting Magnet. IEEE Trans. Appl. Supercond..

[76] Qingmin H., Wenling J., Shuhui Z., Liang R., Ziguang J. Natural Gas Pipeline Leakage Detection Based on FBG Strain Sensor. Proceedings of the 2013 Fifth International Conference on Measuring Technology and Mechatronics Automation.

[77] Han B., Li L., Wu Z., Jing H. Applications of FBG and ZigBee in telemetering of vortex-induced vibration for pipelines. Proceedings of the 2013 22nd Wireless and Optical Communication Conference.

[78] Wang J., Zhao L., Liu T., Li Z., Sun T., Grattan K.T.V. (2017). Novel Negative Pressure Wave-Based Pipeline Leak Detection System Using Fiber Bragg Grating-Based Pressure Sensors. J. Lightw. Technol..

[79] Zhang W., Huang W., Li L., Liu W., Li F. High resolution FBG sensor and its applications in Geophysics. Proceedings of the 2017 16th International Conference on Optical Communications and Networks (ICOCN).

[80] Hu B., Song G., Zhang H., Liu T. A precision edge filter interrogation system for mine Fiber Bragg Grating micro-seismic sensors. Proceedings of the 2017 16th International Conference on Optical Communications and Networks (ICOCN).

[81] Kamikawachi R.C., Abe I., Kalinowski H.J., Fabris J.L., Pinto J.L. Thermal characterization of etched FBG for applications in oil and gas sector. Proceedings of the 2007 SBMO/IEEE MTT-S International Microwave and Optoelectronics Conference.

[82] Kim C.H., Paek I., Yoo N. Monitoring of small wind turbine blade using FBG sensors. Proceedings of the 2010 International Conference on Control, Automation and Systems (ICCAS 2010).

[83] Bremer K., Lewis E., Leen G., Moss B., Lochmann S., Mueller I., Reinsch T., Schroetter J. Fibre optic pressure and temperature sensor for geothermal wells. Proceedings of the 2010 IEEE Sensors.

[84] Huang Q., Zhang C., Liu Q., Ning Y., Cao Y. New type of fiber optic sensor network for smart grid interface of transmission system. Proceedings of the IEEE PES General Meeting.

[85] Campopiano S., Cutolo A., Cusano A., Giordano M., Parente G., Lanza G., Laudati A. (2009). Underwater Acoustic Sensors Based on Fiber Bragg Gratings. Sensors.

[86] Zhang W., Li F., Liu Y., Xiao H. FBG hydrophone: Theory and experiment. Proceedings of the 2008 1st Asia-Pacific Optical Fiber Sensors Conference.

[87] Wang G., Pran K., Sagvolden G., Havsgård G.B., Jensen A.E., Johnson G.A., Vohra S.T. (2001). Ship hull structure monitoring using fibre optic sensors. Smart Mater. Struct..

[88] Allwood G., Wild G., Hinckley S. (2017). Fiber Bragg Grating Sensors for Mainstream Industrial Processes. Electronics.

[89] Zhou Z. Intelligent monitoring technology for machine tools based on FBG sensing. Proceedings of the 2014 International Conference on Innovative Design and Manufacturing (ICIDM).

[90] Pei H., Cui P., Yin J., Zhu H., Chen X., Pei L., Xu D. (2011). Monitoring and warning of landslides and debris flows using an optical fiber sensor technology. J. Mt. Sci..

[91] Allwood G., Hinckley S., Wild G. Optical Fiber Bragg grating based intrusion detection systems for homeland security. Proceedings of the 2013 IEEE Sensors Applications Symposium Proceedings.

[92] Catalano A., Bruno F., Pisco M., Cutolo A., Cusano A. (2014). An Intrusion Detection System for the Protection of Railway Assets Using Fiber Bragg Grating Sensors. Sensors.

[93] Boersma A., Cremers R., Jansen R. (2016). Fiber Bragg Grating Distributed Chemical Sensors. Procedia Eng..

[94] Dziuda L., Krej M., Skibniewski F.W. (2013). Fiber Bragg Grating Strain Sensor Incorporated to Monitor Patient Vital Signs During MRI. IEEE Sens. J..

[95] Witt J., Narbonneau F., Schukar M., Krebber K., Jonckheere J.D., Jeanne M., Kinet D., Paquet B., Depre A., DAngelo L.T. (2012). Medical Textiles With Embedded Fiber Optic Sensors for Monitoring of Respiratory Movement. IEEE Sens. J..

[96] Lee J.R., Ryu C.Y., Koo B.Y., Kang S.G., Hong C.S., Kim C.G. (2003). In-flight health monitoring of a subscale wing using a fiber Bragg grating sensor system. Smart Mater. Struct..

[97] Sante R.D. (2015). Fibre Optic Sensors for Structural Health Monitoring of Aircraft Composite Structures: Recent Advances and Applications. Sensors.

[98] Filograno M.L., Guillen P.C., Rodriguez-Barrios A., Martin-Lopez S., Rodriguez-Plaza M., Andres-Alguacil Á., Gonzalez-Herraez M. (2012). Real-Time Monitoring of Railway Traffic Using Fiber Bragg Grating Sensors. IEEE Sens. J..

[99] Mennella F., Laudati A., Esposito M., Cusano A., Cutolo A., Giordano M., Campopiano S., Breglio G. Railway monitoring and train tracking by fiber Bragg grating sensors. Proceedings of the Third European Workshop on Optical Fibre Sensors.

[100] 3M Science Applied to Life. https://www.3m.com/.

[101] Advanced Optical Solutions GmbH. www.aos-fibre.com.

[102] AFW Technologies Pty Ltd. www.afwtechnologies.com.au.

[103] Alcatel Optronics. http://www.alcatel.com/telecom/optronics/.

[104] Alxenses Company Ltd. www.alxenses.com.

[105] Ascentta Inc. www.ascentta.com.

[106] AtGrating (China). http://www.atgrating.com/en/.

[107] Broptics Technology Inc. www.broptics.com.

[108] DK Photonics Technology Co., Ltd. www.dkphotonics.com.

[109] FBG TECH. www.fbg.co.kr/eng.

[110] FBGS International NV. www.fbgs.com.

[111] Fibreguide Industries. www.fibreguide.com.

[112] Gould Fibre Optic. https://gouldfo.com/.

[113] HBM FibreSensing, S.A. https://www.hbm.com/fs.

[114] InPhoTech. www.inphotech.eu.

[115] ITF Technologies Inc. www.itftechnologies.com.

[116] IXBlue. www.photonics.ixblue.com.

[117] Keysight. www.keysight.com/.

[118] MPB Communications. mpbcommunications.com.

[119] Optoadvance S.r.l. http://www.optoadvance.com/.

[120] Optromix Co. www.optromix.com.

[121] Oxford Lasers Inc. https://www.oxfordlasers.com/.

[122] OZ Optics Limited. http://www.ozoptics.com/.

[123] Proximion AB. www.proximion.com.

[124] Technica Optical Components, LLC. www.technicasa.com.

[125] TeraXion. www.teraxion.com.

[126] Thorlabs Inc. https://www.thorlabs.com/.

[127] VFIBRE Ltd. www.vfibre.com.

